# Supplementary material for: Body Condition in the Tawny Owl Strix aluco near the Northern Limit of Its Range: Effects of Individual Characteristics and Environmental Conditions
Source: Animals (Basel). 2022 Oct 19;12(20):2843. doi: 10.3390/ani12202843 (PMC9597763; doi:10.3390/ani12202843)
Supplement: Supplementary file 1 [file animals-12-02843-s001.zip › Figure S2: Condition index.pdf]

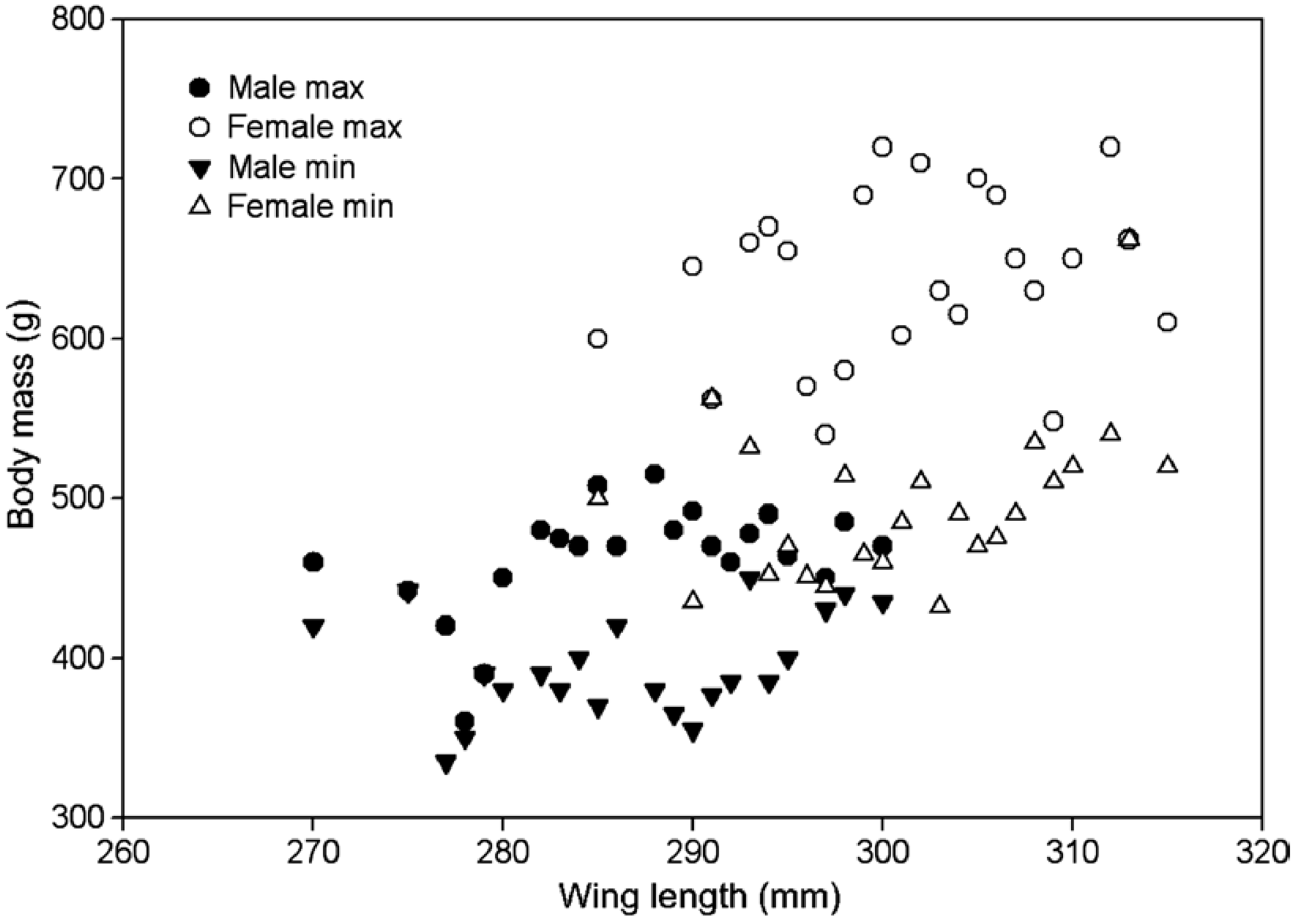

Figure S2. Observed maximum and minimum body mass values (g) in male and female tawny owls of different sizes (indicated by wing length, 22 and 24 size classes (mm) in males and females, respectively) in a population in southern Finland. Linear regression lines fitted to indicate the approximate minimum (min) levels of body mass (BM) in Tawny Owls of different size (wing length, WL) are as follows: $BM_{min} = 1.67*WL - 45.26$ (n = 9) and $BM_{min} = 1.47*WL - 64.22$ (n = 4), for females and males, respectively [9].

By visual inspection of the scatter plots of the body mass vs. wing length distribution, I selected sets of a few extreme values to fit ordinary least squares regression lines to characterise the approximate minimum level of body mass values (g) against size categories (indicated by different wing lengths, mm), separately for females and males [9]. Linear regression lines fitted to indicate the approximate minimum (min) levels of body mass (BM) in Tawny Owls of different size (wing length, WL) were as follows: BM min = 1.67 x WL – 45.26, and BM min = 1.47 x WL – 64.22, for females and males, respectively. The slopes of these lines proved to be roughly similar with those of the regression lines through the respective total samples. The proportions of the deviations of the observed body mass values from the approximated minimum level indicated the condition of the individual measured: (Observed body mass – Minimum body mass)/(Observed body mass).
