# Supplementary material for: Body Condition in the Tawny Owl Strix aluco near the Northern Limit of Its Range: Effects of Individual Characteristics and Environmental Conditions
Source: Animals (Basel). 2022 Oct 19;12(20):2843. doi: 10.3390/ani12202843 (PMC9597763; doi:10.3390/ani12202843)
Supplement: Supplementary file 1 [file animals-12-02843-s001.zip › Figure S3: General vole index.pdf]

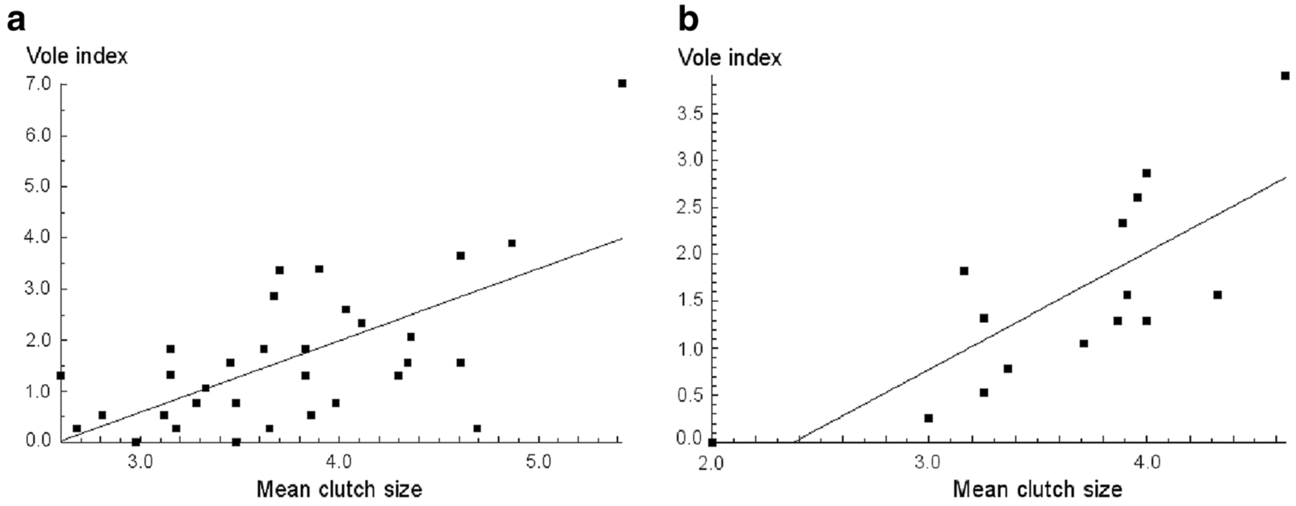

Figure S3. Basics of the general vole index used: The abundance of voles during the breeding season of owls characterized by the results of snap trappings in two local study areas (a and b) near the southern coast of Finland [33]. Voles were caught in both autumn and spring in two periods of time between 1981 and 2012. The longer-term trapping in the study area a covered the whole study period. The smaller-scale trapping effort in the study area b was conducted between 1986 and 2000. In the area a, snap trappings were conducted along four transects. At each line, 16 points of three traps were used, the points located about 25 m from each other, during two 24-h trapping periods (totalling 384 trap nights in each trapping). In the the area b, small mammals were trapped at 30 standard points of three snap traps along a 1.5-km transect (total of 90 traps) throughout a 24-h period. The results (abundance of voles) were expressed as catch indices, indicating the number of individuals caught per 100 trap nights. The annual mean clutch size in a local population of the Tawny Owl *Strix aluco* (x) in relation to the spring abundance of small voles (vole index; y) in southern Finland a in 1981–2012 in our western study area ($y = -3.67 + 1.41x$) and b in 1986–2000 in the study area b ($y = -2.48 + 1.08x$).
