# Supplementary figures and images for: Body Condition in the Tawny Owl Strix aluco near the Northern Limit of Its Range: Effects of Individual Characteristics and Environmental Conditions
Source: Animals (Basel). 2022 Oct 19;12(20):2843. doi: 10.3390/ani12202843 (PMC9597763; doi:10.3390/ani12202843)

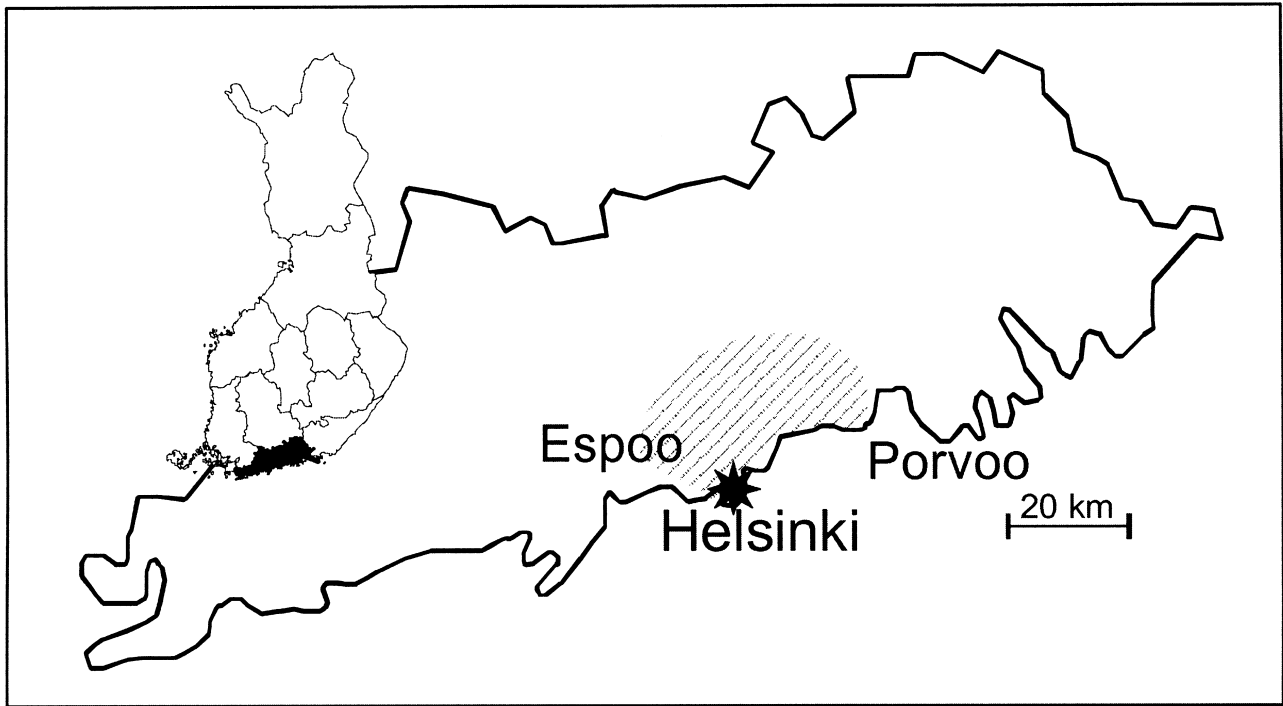

**Figure S1.** The shaded area shows the location of the study area on the southern coast of Finland.

Supplement: Supplementary file 1 [file animals-12-02843-s001.zip › Figure S1. Location of the study area.pdf]
